# Author Correction: A male-drive female-sterile system for the self-limited control of the malaria mosquito *Anopheles gambiae*

**DOI:** 10.1038/s41467-025-66588-w

**Published:** 2025-11-20

**Authors:** Anna Strampelli, Katie Willis, Hannah Robyn Gulliford, Matthew Gribble, Barbara Fasulo, Austin Burt, Andrea Crisanti, Federica Bernardini

**Affiliations:** Department of Life Sciences, Imperial College London, London, UK

**Keywords:** Synthetic biology, CRISPR-Cas9 genome editing, Double-strand DNA breaks

Correction to: *Nature Communications* 10.1038/s41467-025-64489-6, published online 27 October 2025.

In the version of this article initially published, in Fig. 5, the grey lines in the background representing stochastic model simulations were not present and are now amended in the HTML and PDF versions of the article.

