# Supplementary material for: A male-drive female-sterile system for the self-limited control of the malaria mosquito Anopheles gambiae
Source: Nat Commun. 2025 Oct 27;16:9446. doi: 10.1038/s41467-025-64489-6 (PMC12559354; doi:10.1038/s41467-025-64489-6)
Supplement: Supplementary file 1 — Supplementary Information [file 41467_2025_64489_MOESM1_ESM.pdf]

A male-drive female-sterile system for the self-limited control of the malaria mosquito *Anopheles gambiae*

Supplementary Table 1

G1	MDFS (CFP+)	*attP* docking site (GFP+)	Sex assigned in pupae	Sex assigned in adults
1	✓	✓	♂	♂
2	✓	✓	♂	♂
3	✓	✓	♂	♂
4	✓	✓	♂	♂
5	✓	✓	♂	♂
6	✓	X	♂	♀ (intersex)
7	✓	X	♂	♀ (intersex)
8	✓	✓	♂	♀ (intersex)

Supplementary Table 1. G1 transgenics obtained upon injections of the MDFS construct in the *attP* docking site embryos. The docking strain used for the embryo injections mainly consisted of homozygous individuals for the $dsx^{\varphi C31}$ allele. G1 transgenics were expected to be CFP^+ if they contained only the MDFS allele or both CFP^+ and GFP^+ if they contained the MDFS allele and the $dsx^{\varphi C31}$ allele. G1 male number 1, highlighted in light blue, was selected to establish the MDFS strain.

Supplementary Table 2

**Date**	05/23	09/23	12/23	2/24	5/24	1/25
**Transgenic rate**	>95%	100%	100%	>95%	>95%	100%
**MDFS Females intersex**	100%	100%	100%	100%	100%	100%

Supplementary Table 2. Monitoring of the MDFS strain through the rearing practices in our laboratory. The table displays the transgenic rate observed in the progeny of the MDFS strain as well as the rate of the intersex phenotype observed in MDFS females. The transgenic rate for the MDFS strains ranged from over 95% to 100%, and all MDFS females consistently exhibited the intersex phenotype. Routine analyses are conducted on approximately 200 individuals.

Supplementary Figure 1

A

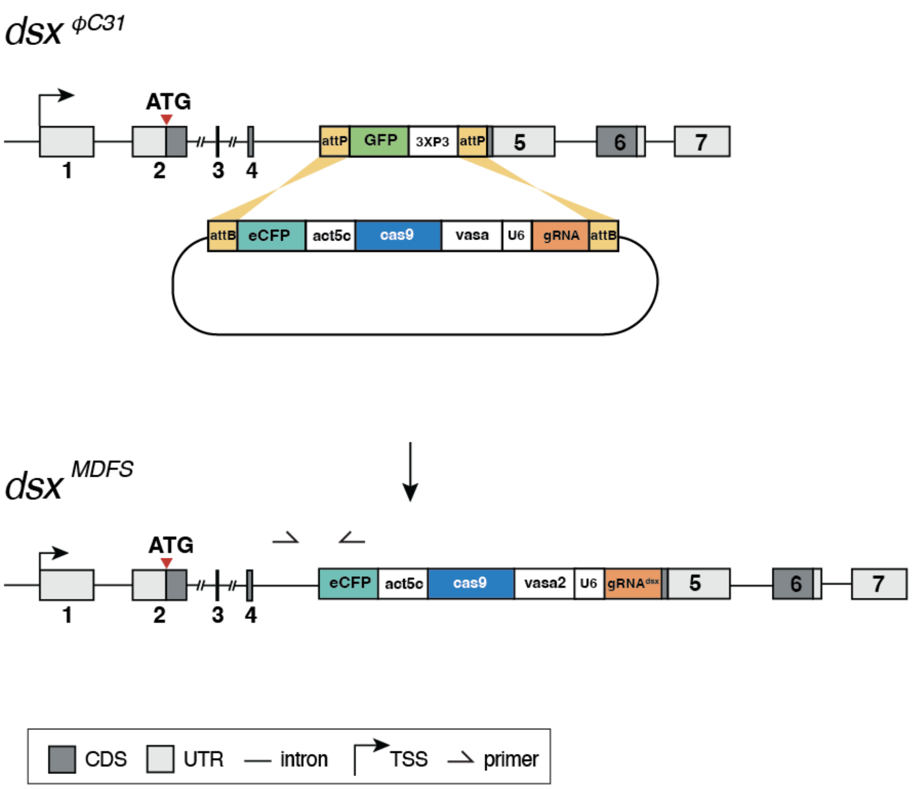

B

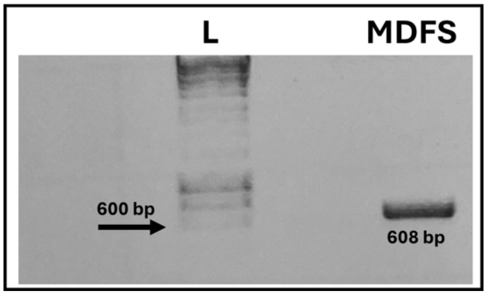

Supplementary Figure 1. Recombinase-mediated cassette exchange was used to swap out the *dsx*^{φC31} allele with the MDFS construct. (**A**) The catalytic activity of the φC31 integrase was used to replace the *dsx*^{φC31} allele, which contains the 3xP3::GFP transcription unit surrounded by *attP* sites, with the MDFS construct. The MDFS construct consists of an *actin5c::eCFP* fluorescent marker, a *Cas9* driven by the germline *vasa2* promoter and a *U6::gRNA*^{dsxF} cassette targeting *dsx* at the intron 4-exon 5 boundary, all surrounded by two *attB* sites. At the bottom panel, the schematic depicts the MDFS construct within the *dsx* gene post-integration. The insertion of the construct hinders the production of functional *dsxF* transcript, leaving *dsxM* unaffected. Non-coding regions (UTR) are shaded in light grey, coding regions (CDS) are shaded in dark grey, and black lines indicate introns and are not in scale. The bent arrow denotes the transcription start site (TSS) of the *dsx* gene. (**B**) PCR analysis performed to confirm the MDFS construct's correct integration into the *dsx* gene. Primers localisation in (A). See the Methods section for information on the primers used.

Supplementary Figure 2

A

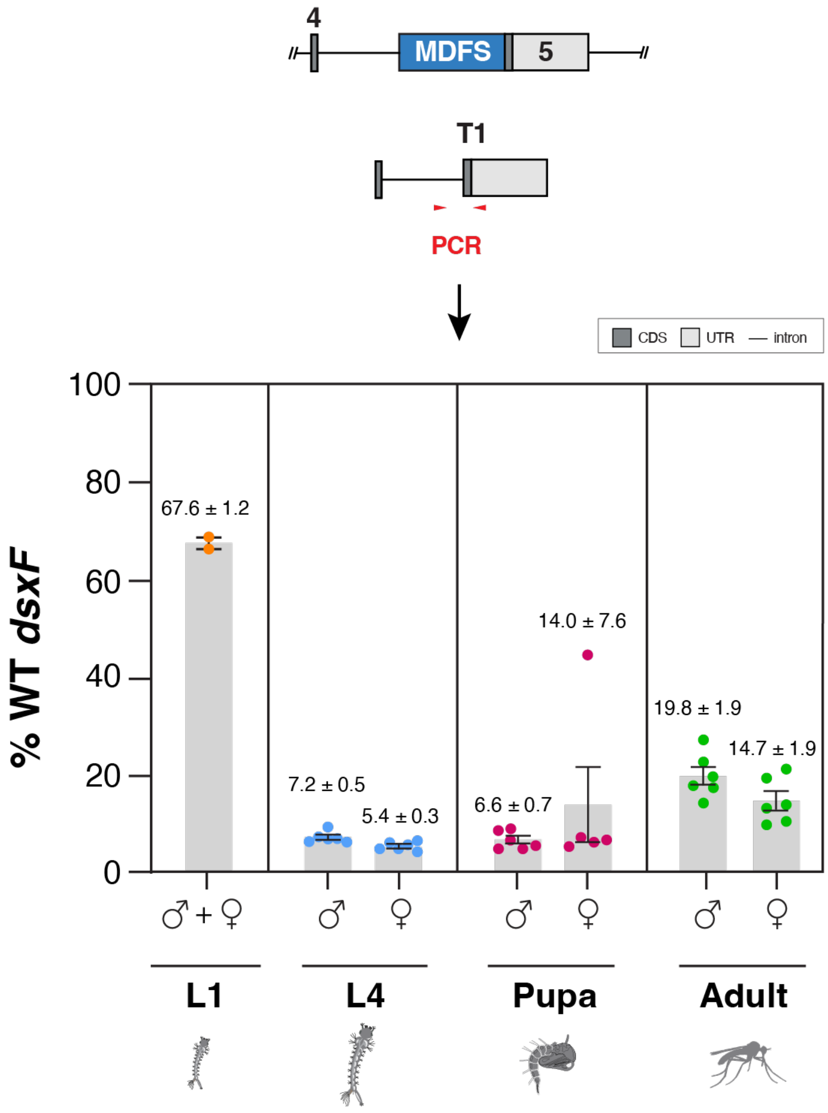

B

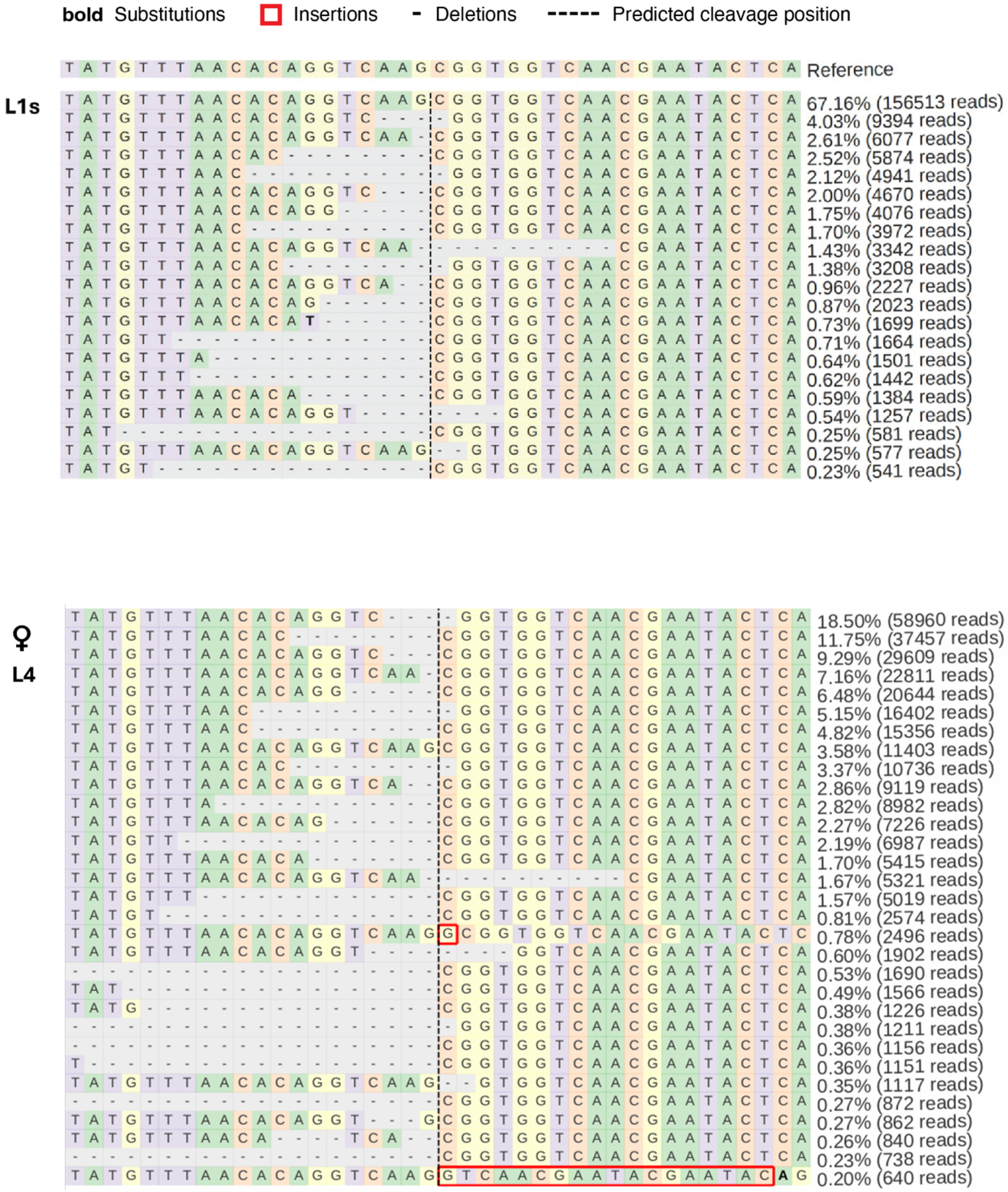
bold Substitutions
Insertions
- Deletions
----- Predicted cleavage position
Reference
L1s
67.16% (156513 reads)
4.03% (9394 reads)
2.61% (6077 reads)
2.52% (5874 reads)
2.12% (4941 reads)
2.00% (4670 reads)
1.75% (4076 reads)
1.70% (3972 reads)
1.43% (3342 reads)
1.38% (3208 reads)
0.96% (2227 reads)
0.87% (2023 reads)
0.73% (1699 reads)
0.71% (1664 reads)
0.64% (1501 reads)
0.62% (1442 reads)
0.59% (1384 reads)
0.54% (1257 reads)
0.25% (581 reads)
0.25% (577 reads)
0.23% (541 reads)
♀
L4
18.50% (58960 reads)
11.75% (37457 reads)
9.29% (29609 reads)
7.16% (22811 reads)
6.48% (20644 reads)
5.15% (16402 reads)
4.82% (15356 reads)
3.58% (11403 reads)
3.37% (10736 reads)
2.86% (9119 reads)
2.82% (8982 reads)
2.27% (7226 reads)
2.19% (6987 reads)
1.70% (5415 reads)
1.67% (5321 reads)
1.57% (5019 reads)
0.81% (2574 reads)
0.78% (2496 reads)
0.60% (1902 reads)
0.53% (1690 reads)
0.49% (1566 reads)
0.38% (1226 reads)
0.38% (1211 reads)
0.36% (1156 reads)
0.36% (1151 reads)
0.35% (1117 reads)
0.27% (872 reads)
0.27% (862 reads)
0.26% (840 reads)
0.23% (738 reads)
0.20% (640 reads)

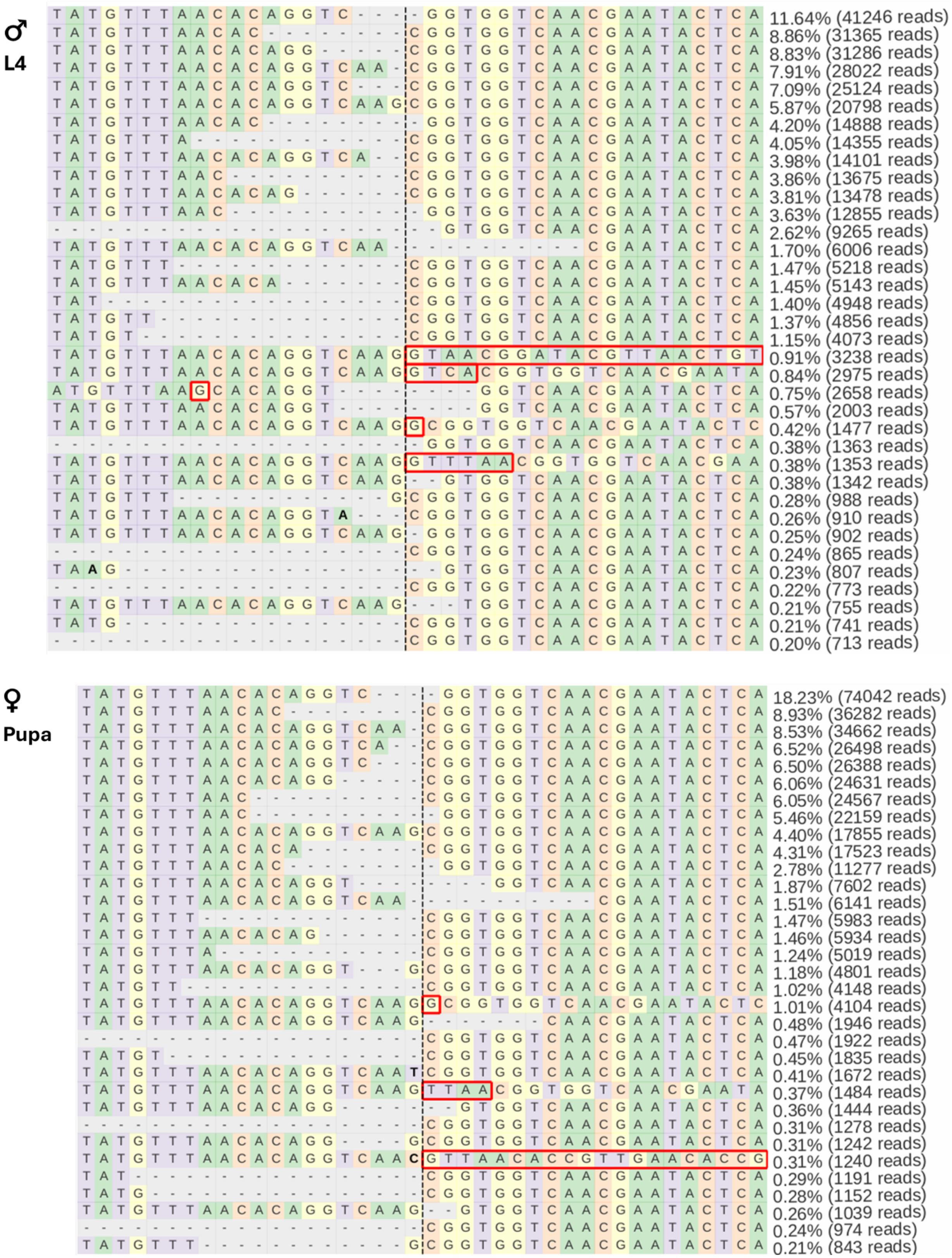
♂
L4
11.64% (41246 reads)
8.86% (31365 reads)
8.83% (31286 reads)
7.91% (28022 reads)
7.09% (25124 reads)
5.87% (20798 reads)
4.20% (14888 reads)
4.05% (14355 reads)
3.98% (14101 reads)
3.86% (13675 reads)
3.81% (13478 reads)
3.63% (12855 reads)
2.62% (9265 reads)
1.70% (6006 reads)
1.47% (5218 reads)
1.45% (5143 reads)
1.40% (4948 reads)
1.37% (4856 reads)
1.15% (4073 reads)
0.91% (3238 reads)
0.84% (2975 reads)
0.75% (2658 reads)
0.57% (2003 reads)
0.42% (1477 reads)
0.38% (1363 reads)
0.38% (1353 reads)
0.38% (1342 reads)
0.28% (988 reads)
0.26% (910 reads)
0.25% (902 reads)
0.24% (865 reads)
0.23% (807 reads)
0.22% (773 reads)
0.21% (755 reads)
0.21% (741 reads)
0.20% (713 reads)
♀
Pupa
18.23% (74042 reads)
8.93% (36282 reads)
8.53% (34662 reads)
6.52% (26498 reads)
6.50% (26388 reads)
6.06% (24631 reads)
6.05% (24567 reads)
5.46% (22159 reads)
4.40% (17855 reads)
4.31% (17523 reads)
2.78% (11277 reads)
1.87% (7602 reads)
1.51% (6141 reads)
1.47% (5983 reads)
1.46% (5934 reads)
1.24% (5019 reads)
1.18% (4801 reads)
1.02% (4148 reads)
1.01% (4104 reads)
0.48% (1946 reads)
0.47% (1922 reads)
0.45% (1835 reads)
0.41% (1672 reads)
0.37% (1484 reads)
0.36% (1444 reads)
0.31% (1278 reads)
0.31% (1242 reads)
0.31% (1240 reads)
0.29% (1191 reads)
0.28% (1152 reads)
0.26% (1039 reads)
0.24% (974 reads)
0.21% (843 reads)

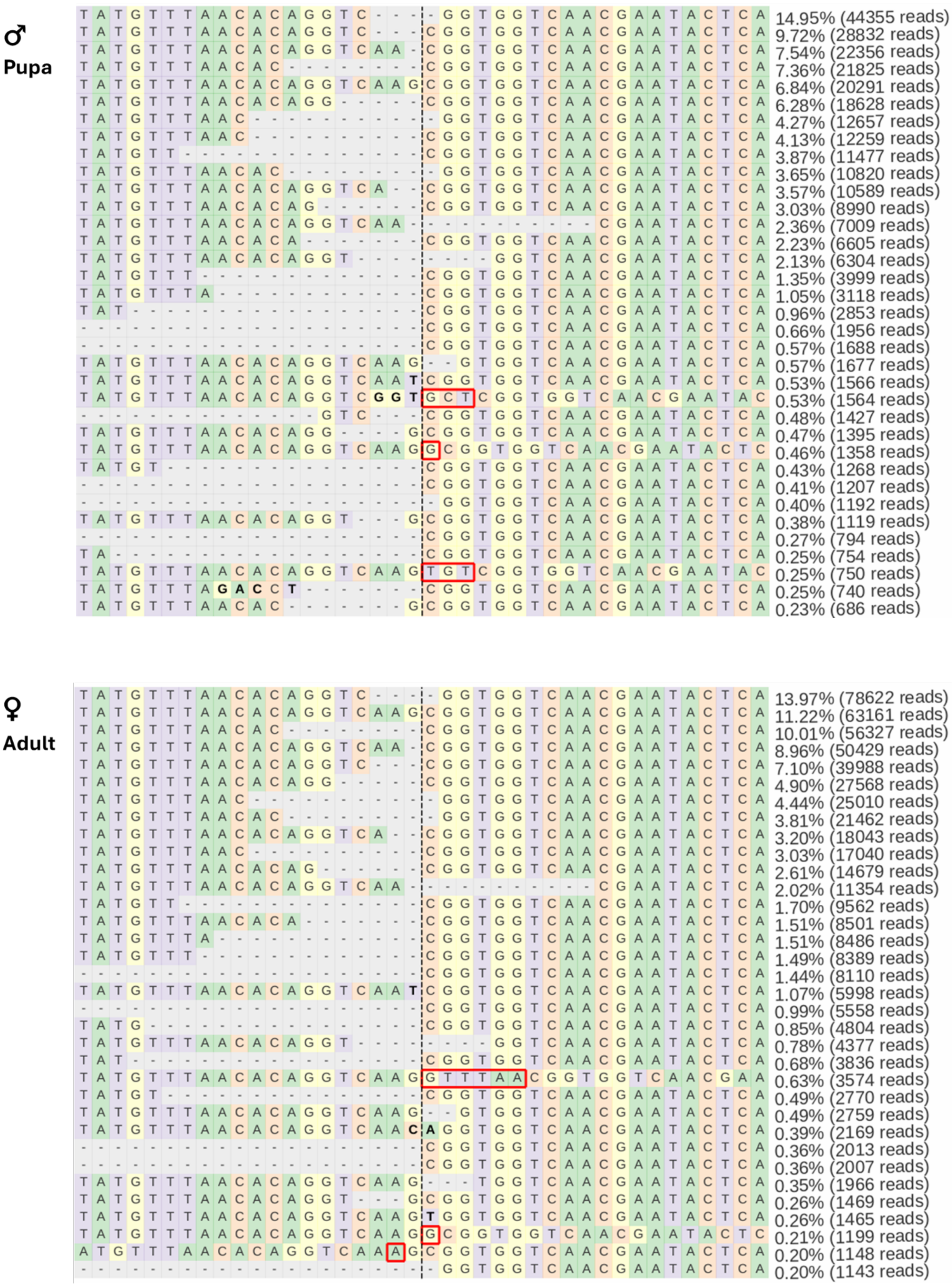
♂
Pupa
14.95% (44355 reads)
9.72% (28832 reads)
7.54% (22356 reads)
7.36% (21825 reads)
6.84% (20291 reads)
6.28% (18628 reads)
4.27% (12657 reads)
4.13% (12259 reads)
3.87% (11477 reads)
3.65% (10820 reads)
3.57% (10589 reads)
3.03% (8990 reads)
2.36% (7009 reads)
2.23% (6605 reads)
2.13% (6304 reads)
1.35% (3999 reads)
1.05% (3118 reads)
0.96% (2853 reads)
0.66% (1956 reads)
0.57% (1688 reads)
0.57% (1677 reads)
0.53% (1566 reads)
0.53% (1564 reads)
0.48% (1427 reads)
0.47% (1395 reads)
0.46% (1358 reads)
0.43% (1268 reads)
0.41% (1207 reads)
0.40% (1192 reads)
0.38% (1119 reads)
0.27% (794 reads)
0.25% (754 reads)
0.25% (750 reads)
0.25% (740 reads)
0.23% (686 reads)
♀
Adult
13.97% (78622 reads)
11.22% (63161 reads)
10.01% (56327 reads)
8.96% (50429 reads)
7.10% (39988 reads)
4.90% (27568 reads)
4.44% (25010 reads)
3.81% (21462 reads)
3.20% (18043 reads)
3.03% (17040 reads)
2.61% (14679 reads)
2.02% (11354 reads)
1.70% (9562 reads)
1.51% (8501 reads)
1.51% (8486 reads)
1.49% (8389 reads)
1.44% (8110 reads)
1.07% (5998 reads)
0.99% (5558 reads)
0.85% (4804 reads)
0.78% (4377 reads)
0.68% (3836 reads)
0.63% (3574 reads)
0.49% (2770 reads)
0.49% (2759 reads)
0.39% (2169 reads)
0.36% (2013 reads)
0.36% (2007 reads)
0.35% (1966 reads)
0.26% (1469 reads)
0.26% (1465 reads)
0.21% (1199 reads)
0.20% (1148 reads)
0.20% (1143 reads)

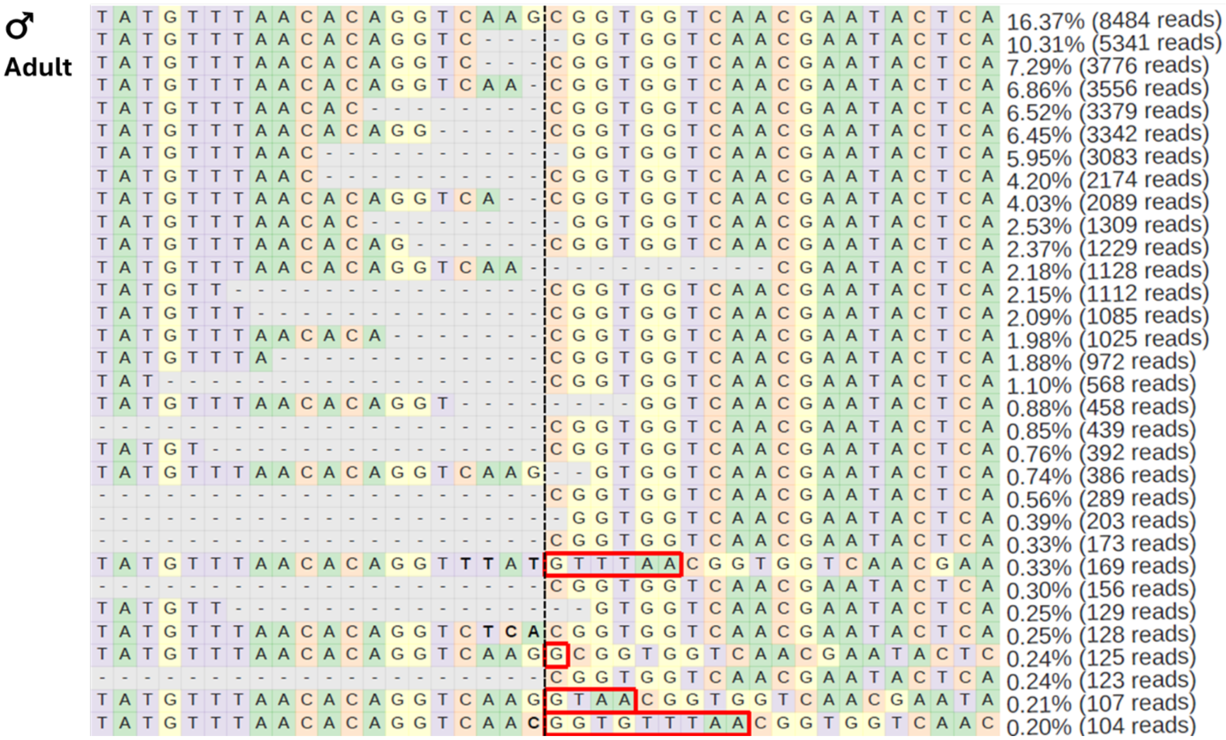

Supplementary Figure 2. Pooled amplicon sequencing at the *dsxF* target site. **(A)** Frequency of wild-type alleles at the *dsxF* target site in MDFS individuals at different developmental stages. We performed a pooled amplicon sequencing analysis at the *dsxF* target site (T1) to quantify the level of disruption at different life stages of male and female MDFS individuals. The wild-type (WT) *dsxF* frequency refers to the allele that does not contain the MDFS transgene (the PCR symbol is placed at the top of the diagram to symbolise the region amplified). In line with this, the percentage of WT alleles shown in the figure is relative to end-joining alleles rather than all (i.e., the MDFS alleles are not included). L1 samples were two pools of >100 larvae, while each dot represents a single individual for the other stages. Since L4 male and female larvae are phenotypically indistinguishable, and MDFS female pupae are phenotypically male-like, to separate males and females at these two developmental stages, we crossed MDFS males to females homozygous for an mCherry marker under the male-specific *β2* promoter. The male

progeny of this cross expressed mCherry in the testes, and the female progeny did not, which allowed the two sexes to be separated for the analysis. Horizontal bars indicate the mean and the s.e.m. Figure was adapted from: iStock.com/LCOSMO. **(B)** Representative examples of the sequencing data and repair outcomes observed for each analysed group. Each image represents the sequencing outcomes from one sample (dot) shown in (A) (i.e., pooled L1 males and females or L4 single male or female, pupae or adults). A cut-off of 0.20% frequency was set to improve the readability of the results. Raw amplicon sequencing data are available at SRA (NCBI). Accession code: PRJNA1227481.

Supplementary Figure 3

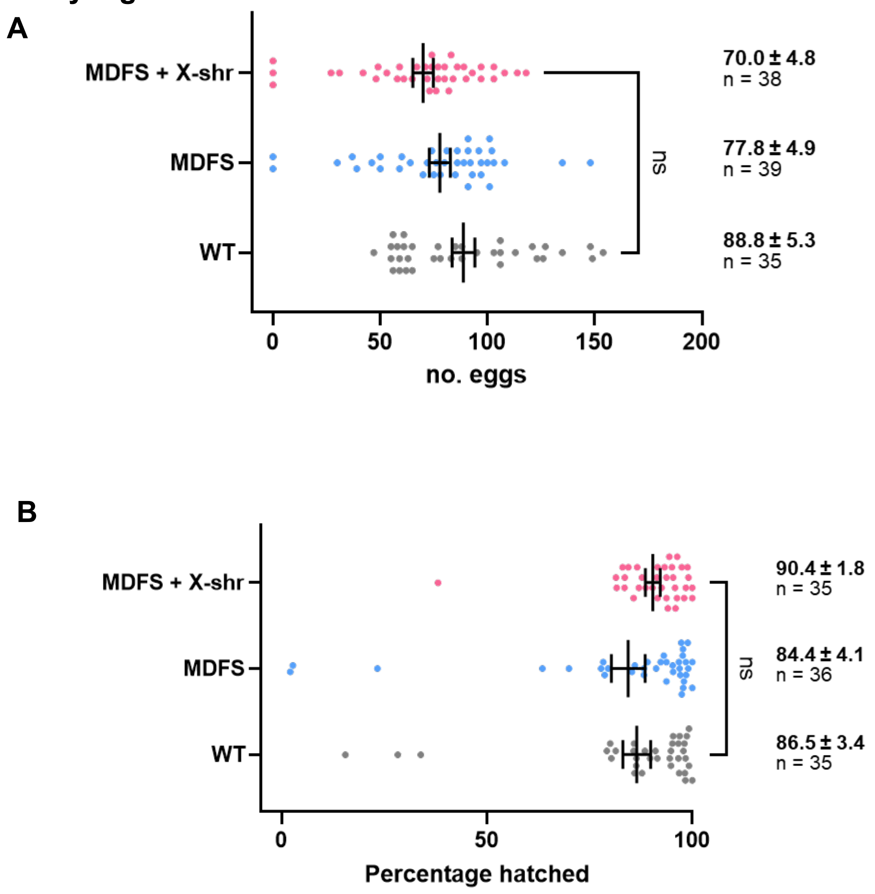

Supplementary Figure 3. Fertility assay of MDFS and MDFS + X-shredder males. **(A)** The egg output and **(B)** hatching rate (larvae/eggs) were measured and compared to those of wild-type (WT) controls. No significant differences ('ns') were found among these genotypes (P>0.05; Kruskal-Wallis test adjusted for multiple comparisons). Vertical bars indicate the mean and the s.e.m.. Source data are provided as a Source Data file.

Supplementary Figure 4

A

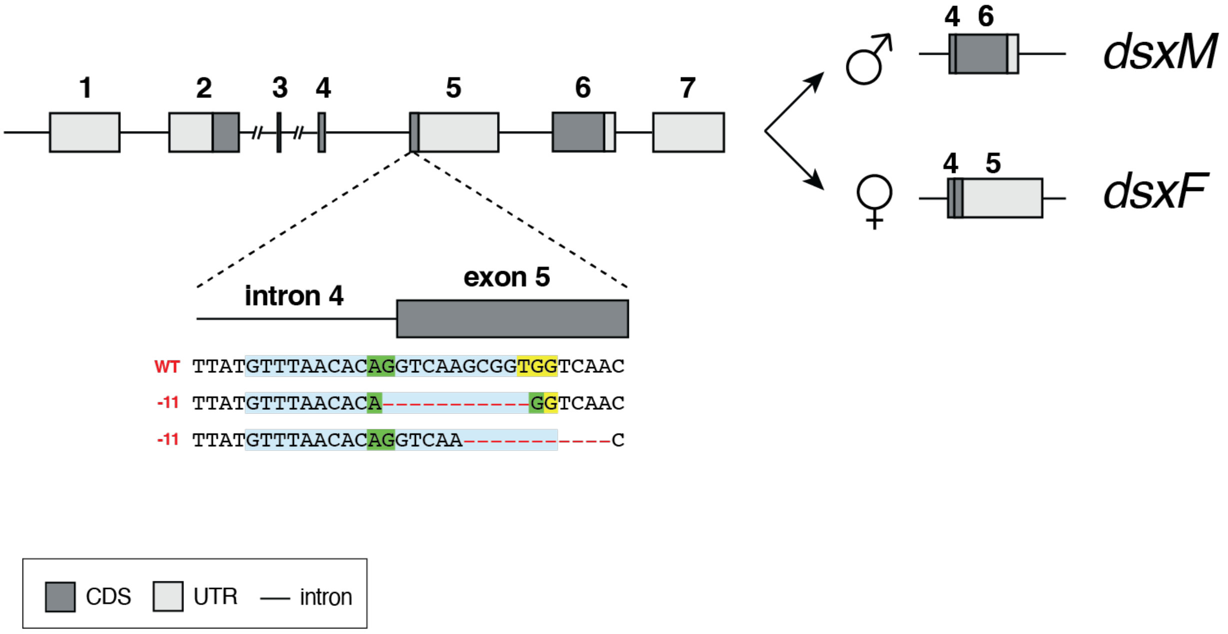

B

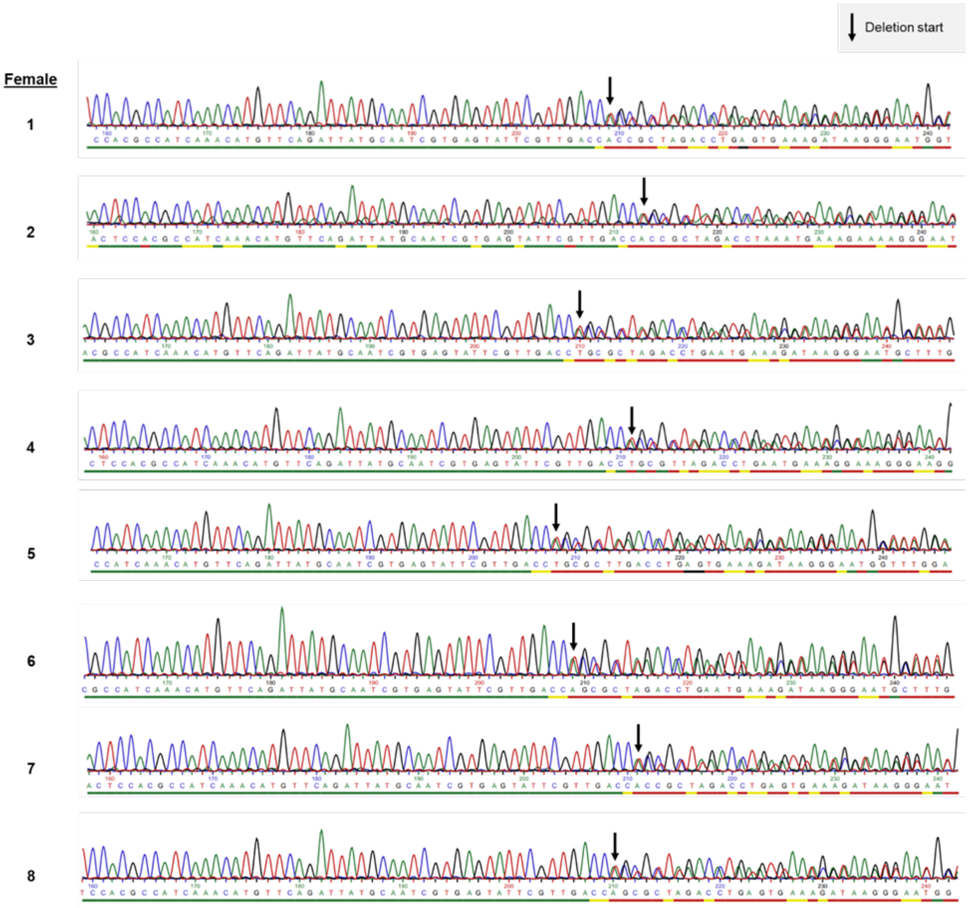

Supplementary Figure 4. Deletions identified in the eight mosaic females that did not inherit the MDFS allele (CFP-). **(A)** The 11 bp deletion aligned against the wildtype allele, which could be portrayed in one of two ways because it resulted from microhomology-mediated end joining (MMEJ) repair. The AG sequence of the splice acceptor site is retained and highlighted in green, and the PAM is highlighted in yellow. **(B)** The electropherograms showing the Sanger sequences of the *dsxF* target site in the females analysed, where the 11-bp deletion begins at the transition from one to double peaks and is indicated by the black arrow. Note that the sequencing was performed with a reverse primer with respect to the *dsxF* sequence, so the “GTTGACC” sequence

that is 5’ of the deletion in (A) is the reverse complement of the “GGTCAAC” sequence that is 3’ of the deletion in (B).

Supplementary Methods

1. Modelling

The following sections describe the mathematical models and computer simulations used in this study. All modelling was performed using The Julia Computing language and is available on GitHub via https://github.com/KatieWillis/MDFS.

1.1. Deterministic model

Summary

To model the impact of releasing different genetic constructs into a wild population we developed two deterministic models based on the structure developed by Burt and Deredec (2018)[1]. Both models simulate a randomly mating population of infinite size through time using discrete, non-overlapping generations, two sexes (males and females), and two life stages (juveniles and adults). Juvenile survival is density-dependent according to the Beverton-Holt model such that the probability of surviving is $\theta\left(\frac{\alpha}{\alpha+Z}\right)$, where θ is the density-independent probability the juvenile survives to adulthood, α determines the strength of density-dependent mortality and Z is the number of juveniles in the population. Since we report relative population sizes (compared to the pre-release equilibrium), the results are unaffected by the precise value of α. During each generation females produce f fertilised eggs, with the number of males in the population not affecting production, and therefore the intrinsic rate of increase (R_m) of the wild-type population is $\frac{f\,\theta}{2}$. For all simulations we assume an Rm of 6, following that of Deredec *et al* 2011[2].

MDFS model

To model the MDFS construct alone (without the X-shredder) we include a single locus with four alleles where A1 is the wildtype allele, A2 is the MDFS construct inserted into and disrupting the wild type allele, A3 and A4 are cleavage-resistant recessive and dominant alleles produced by non-homologous end joining. We therefore track 10 male and 10 female genotypes. Depending on the genotype, i.e. whether the genotype contains an MDFS construct and target site, the probability of gamete transmission may be altered due to homing or NHEJ. In individuals carrying the MDFS and WT alleles, cleavage of a WT occurs with probability c, after which repair can occur by NHEJ with probability j converting the WT to a dominant or recessive cleavage-resistant allele with probabilities p or $1-p$ respectively. Alternatively, repair can occur through homology-directed repair, resulting in homing with probability $1 - j$, converting the WT target site to the MDFS construct. Supplementary Table 3 summarises parameters used to model inheritance.

To model the fitness effects due to disruption of the locus (either by construct insertion or NHEJ mutation) a series of sex-specific (X; male or female) parameters were used. The fitness of wild-type homozygotes is standardised to one; and the fitness of genotypes homozygous for the construct A2/A2 or dominant cleavage resistant allele A3/A3 is $1 - s_{\alpha}^{X}$; homozygous for the recessive cleavage resistant allele A4/A4 is $1 - s_{\mathrm{a}}^{X}$; heterozygous for the construct A1/A2 or dominant cleavage resistant allele A1/A3 is $1 - h_{\alpha}^{X} s_{\alpha}^{X}$; heterozygous for the recessive cleavage resistant allele A1/A4 is $1 - h_{a}^{X} s_{a}^{X}$, or heterozygous carrying two of any of the construct, dominant or recessive cleavage-resistant alleles A2/A3 or A2/A4 or A3/A4 is $1 - s_{a}^{X}$, where s_{α}^{X},s_{a}^{X} and $s_{a\alpha}^{X}$ are selection coefficients and h_{α}^{X} and h_{a}^{X} are dominance coefficients. For all simulations, we assume that the insertion locus is essential for survival and that fitness costs cause lethality after density-dependent juvenile mortality (e.g., as if pupae die or adults survive but are both infertile

and unable to bite/transmit disease). We also assume that all resistant alleles produced through NHEJ were non-functional (with either recessive or dominant costs), and that functional resistance was not possible. This sex-specific parameterisation of the fitness parameters also allowed for modelling of a series of alternative self-limiting strategies (Supplementary Table 4).

Supplementary Table 3. Inheritance parameters for the MDFS and X-shredder constructs.

Parameter	Description	Data source	Idealised value	Empirica l value
**d**	Proportion of offspring carrying the mutation	Experiment	-	0.9955
**u**	Proportion of non-homed chromosomes which are NHEJ	Hammond et al 2021[3]	-	0.392
**e**	Probability of homing	$2d-1$	-	0.991
**c**	Probability of cleavage	$e+(1-e)u$	1	0.995
**j**	Probability of NHEJ given joining	$\frac{(1-e)u}{e+(1-e)u}$	0	0.00315
**p**	Proportion of NHEJ products which are dominant	Unknown	0	0.0 (variable)
**m**	Proportion of Y-bearing sperm produced by X-shredder males	Experiment	1	0.95
**r**	Recombination rate between MDFS and X-shredder	10Mb distance between MDFS and XS loci. An estimated 1cM/Mb for *An. Gambiae* Pombi et al 2006[4]	-	0.1

Supplementary Table 4. Model fitness parameters

Fitness costs		MDFS	SIT and RIDL	fsRIDL	XS
s_{α}^{X}	Fitness cost for the construct or dominant NHEJ product	1 F	1	1 F	-
s_{a}^{X}	Fitness cost for the recessive NHEJ product	1 F	-	-	-
h_{α}^{X}	Dominance coefficient for the construct or dominant NHEJ product	1 F	1	1 F	-
h_{a}^{X}	Dominance coefficient for the recessive NHEJ product	0	-	-	-
$s_{a\alpha}^{X}$	Fitness cost for heterozygous for the two types of disrupted allele	1 F	-	-	-

The superscript F indicates the case where fitness costs are applied only to females and the value in males is zero.

MDFS and X-shredder model

We next extended to single locus model to include a second locus with two alleles, where B1 is the wild type and B2 is the construct carrying an X-shredder inserted into a neutral locus. The impact of the MDFS on gamete transmission is modelled as in the first model. In addition, for males carrying at least one X-shredder, sperm is produced carrying Y or X chromosomes at a ratio of $m: 1-m$. Linkage between loci is modelled by assuming the two loci are on a single chromosome and recombination can occur with probability r. Here, loci on different chromosomes can be modelled with $r = 0.5$. Since we allow the option for linkage between the two loci, we track 8 haplotypes and therefore 36 male and 36 female genotypes. Fitness effects were modelled as in the first model, assuming that the B locus was neutral, and that insertion of the X-shredder had no impact on fitness.

Analysis of simulations

For all MDFS simulations released males were heterozygous for the MDFS and X-shredder construct. When released in the same males, the MDFS and X-shredder were assumed to be on separate chromosomes. Allele frequencies were censused at the adult stage, after fitness costs had been applied. The release rate required to suppress the relative number of females by a certain percentage within a certain number of releases was calculating by simulating releases of 0.1% and incrementing the release rate by 0.1% until the desired level of suppression had been achieved within the release period.

1.1. Stochastic cage trial simulation

Summary

We next developed a stochastic model capable of simulating populations in discrete generations mirroring the experimental cage trial design. To do so we model a single locus with 3 alleles where the first allele (A1) is the wildtype, the second (A2) is the MDFS construct inserted into and disrupting the wildtype allele and the third (A3) is a cleavage resistant allele with recessive fitness costs. Here the population genetics of the model is a simplified version of the single-locus deterministic model that includes 3 alleles, rather than 4, by making a conservative assumption that all NHEJ mutations are recessive. The model therefore tracks 6 male and 6 female genotypes through each generation (A1A1, A1A2, A1A3, A2A2, A2A3 and A3A3 for both males and females).

Adult population and releases

We began each simulation with 200 WT male and 200 WT female pupae and performed the following steps each generation. First the number of adult mating females $\left(A_i^F(t)\right)$ of each genotype in generation t was sampled from a binomial distribution, $A_i^F(t) \sim Bin\left(P_i^F(t), M^F\right)$, where $P_i^F(t)$ is the number of female pupae of the focal genotype i in generation t and M^F is the

probability of females reaching reproductive maturity. To account for the dominant fitness cost associated with the MDFS construct and the recessive costs of the NHEJ mutation, the number of A1A2, A2A3 and A3A3 females was set to zero, assuming none of these females reached reproductive maturity. Next, the number of adult mating males of each genotype were obtained. In a release generation, 100 A1A2 male pupae (heterozygotes for MDFS and WT alleles) were first added to the existing male pupae population. Next the number of adult mating males $\left(A_i^M(t)\right)$ of each genotype i in generation t was sampled from a binomial distribution, $A_i^M(t) \sim Bin\left(P_i^M(t), M^M\right)$, where $P_i^M(t)$ is the number of male pupae of the focal genotype i in generation t (after any releases have been made) and M^M is the probability of males surviving until reproductive maturity. No fitness costs were added for males since they were assumed to be of equal fitness to the wild type.

Mating and zygote production

To simulate mating, the number of mating females of each genotype mated by a male of each genotype was generated, assuming males and females mated at random, that each female mates only once and that the number of males is not limiting to the formation of mating pairs. To do so, for each of the six female genotypes, the numbers of mating adult females of genotype i mated to males of each genotype $1 \dots j$ in generation t ($A_{(i,1 \dots j)}^F M(t)$) was obtained by sampling from a multinomial distribution, $A_{(i,1 \dots j)}^F M(t) \sim M\left(A_i^F(t), a_{(1 \dots j)}^M(t)\right)$ where $A_i^F(t)$ is the number of mated adult females of genotype i in generation t and $a_{1 \dots j}^M$ is the frequency of males of each of the six male genotypes (1 to j). For each individual mated female, the number of eggs produced (o) was randomly drawn from the list of egg numbers (E) obtained empirically (Supplementary Table 5). The number of eggs fertilised by sperm to generate each zygote genotype ($1 \dots k$) of both sexes combined was calculated by sampling from a multinomial distribution, $Z_{i,j,1 \dots k}^{F+M} \sim M\left(o, I_{i,j,1 \dots k}\right)$ where

o is the number of eggs produced by the focal mated female and $I_{i,j,k}$ is the expected proportion of zygote genotype k produced based on the genotype of mother i, and father j making up the mating pair. The expected proportion ($I_{i,j,k}$) takes into consideration biases in gamete transmission caused by homing in MDFS and WT heterozygote parents, which is modelling similarly to the single-locus deterministic model. To calculate $I_{i,j,k}$ we assume that, prior to segregation of alleles to form gametes, in individuals carrying the MDFS (A2) and WT (A1) alleles cleavage of a WT occurs with probability c, after which repair can occur by NHEJ with probability j converting the WT to a recessive cleavage-resistant allele. Alternatively, repair an occur through HDR, resulting in homing with probability $1 - j$, converting the WT target site to the MDFS construct. We then assume random segregation of alleles in both males and females after any modification due to homing and/or mutation had been considered. This process was parameterised using empirical data in the same way as the deterministic model (Supplementary Table 3). Supplementary Table 5 summarises the additional parameters used to model the dynamics of the cage population. The zygotes produced were assigned a sex at random assuming an equal sex ratio, with the number of male zygotes of genotype j in generation $t+1$ ($Z_j^M(t+1)$) being sampled from a binomial distribution, $Z_j^M(t+1) \sim Bin(Z_j(t+1), 0.5)$, where $Z_j(t+1)$ is the total number of zygotes of genotype j in both sexes in the population at generation $t+1$. The number of female zygotes of genotype j at generation $t+1$ ($Z_j^F(t+1)$) was then $Z_j^F(t+1) \sim Z_j(t+1) - Z_j^M(t+1)$.

Zygote maturation and sampling

The number of larvae of each sex (X) and genotype (j) which successfully hatched in generation $t+1$ $\left(L_j^X(t+1)\right)$ was generated by sampling from a binomial distribution, $L_j^X(t+1) \sim Bin\left(Z_j^X(t+1), h\right)$, where h is the hatching rate. To measure the frequency of MDFS in the larvae, 750 larvae

were sampled from the population by randomly sampling without replacement from a vector containing variables representing each of the 12 sex-specific genotypes present at the frequency that each was present in the current population. The frequency of MDFS was calculated from the population of 750 individuals. If less than 750 larvae were present, the MDFS frequency was calculated using all larvae. To generate the pupal population, first artificial mortality was applied to the total larval population by down-sampling, selecting 1/4 larvae at random, following the same random sampling method used to obtain the MDFS larval frequency described above. Then the number of larvae surviving to pupation of sex X and genotype j in generation $t+1$ ($P_j^X(t+1)$) was sampled from a binomial distribution, $P_j^X(t+1) \sim Bin(L_j^X(t+1), l)$, where l is the larval survival rate. 400 pupae were sampled from the population following the same sampling method as for calculating the MDFS frequency. Where there were less than 400 pupae, all were used to seed the next generation. For each simulation male releases began at the 4th generation and were released every generation thereafter or ceased after 3 releases.

Supplementary Table 5. Cage population dynamics parameters.

Parameter	Description	Data source	Value
E	Observed number of eggs laid by females in laboratory conditions	Phenotype assay	127, 113, 55, 61, 61, 95, 58, 88, 77, 65, 59, 78, 75, 149, 123, 83, 106, 47, 154, 126, 59, 85, 62, 88, 121, 135, 148, 106, 103, 65, 56, 106, 56, 63, 55, 37, 59, 92, 100, 108, 50, 97, 46, 78, 86, 81, 72, 101, 70, 93, 0, 91, 86, 91, 76, 91, 101, 39, 135, 75, 80, 103, 0, 60, 64, 50, 30, 96, 102, 89, 74, 97, 148, 85, 58, 49, 103, 97, 48, 0, 72, 71, 59, 27, 82, 80, 86, 61, 84, 53, 114, 76, 74, 90, 80, 0, 103, 89, 108, 67, 65, 74, 77, 31, 77, 118, 83, 42, 73, 0, 97, 93, 145, 99, 171, 87, 154, 157, 177, 78, 140, 154,

			194, 119, 48, 128, 149, 182, 125, 96, 150, 221, 0, 146, 149, 54, 37, 191, 0, 174, 65, 163, 119, 143, 138, 157, 75, 0, 154, 104, 144, 139, 155, 148, 167, 152, 0, 139, 147, 115, 0, 156, 121, 113, 142, 151, 95, 160, 169, 136, 145, 164, 96, 0, 82, 115, 132, 121, 132, 92, 141, 69, 0, 0, 0, 100, 89, 64, 140, 0, 77, 0, 101, 128, 82
h	Hatching rate	Phenotype assay	0.862
l	Larvae survival	Phenotype assay	0.96
p	Pupae emergence	Phenotype assay	0.9915
b	Survival to feeding	Phenotype assay	0.937
m^F	Female mating probability	Phenotype assay	0.933
m^M	Males surviving to mating (those which did not drown)	Phenotype assay	0.9356
M^F	Probability of female pupae surviving to mating	$p\ b\ m^F$	0.8668
M^M	Probability of male pupae surviving to mating	$p\ b\ m^m$	0.8692

References

1. BURT, A. & DEREDEC, A. 2018. Self-limiting population genetic control with sex-linked genome editors. *Proc Roy Soc Lond B,* 285.

2. DEREDEC, A., GODFRAY, H. C. J. & BURT, A. 2011. Requirements for effective malaria control with homing endonuclease genes. *Proc Natl Acad Sci,* 108**,** E874-E880.

3. HAMMOND, A., KARLSSON, X., MORIANOU, I., KYROU, K., BEAGHTON, A., GRIBBLE, M., KRANJC, N., GALIZI, R., BURT, A., CRISANTI, A. & NOLAN, T. 2021. Regulating the expression of gene drives is key to increasing their invasive potential and the mitigation of resistance. *PLOS Genet,* 17**,** e1009321.

4. POMBI M, STUMP AD, DELLA TORRE A, BESANSKY NJ. Variation in recombination rate across the X chromosome of Anopheles gambiae. *Am J Trop Med Hyg.* 2006 Nov;75(5):901-3. PMID: 17123984.
